# Getting more light into the dark room of editorial conflicts of interest

**DOI:** 10.7189/jogh.08.010101

**Published:** 2018-06

**Authors:** Ana Marušić, Rafael Dal-Ré

**Affiliations:** 1 *Journal of Global Health* and Department of Research in Biomedicine and Health, University of Split School of Medicine, Split, Croatia; 2 Epidemiology Unit, Health Research Institute-Fundación Jiménez Díaz University Hospital, Universidad Autónoma de Madrid, Madrid, Spain

A current standard in research is declaration of financial and nonfinancial conflicts of interest (CoI) related to the research process and the publication of results. However, policies on disclosing researchers’ CoI introduced by research funders, such as the National Institutes of Health (NIH) in the USA, do not work in practice [1] and there are calls for the creation of publicly accessible registries of researchers’ CoI [2]. Journals have also developed CoI policies for authors, who present the results of their research in journals, and for peer reviewers, who participate in the evaluation of the work submitted to the journal [3,4].

What about journal editors, who may also have competing interests in relation to submitted manuscripts and even be authors on manuscripts submitted to their own journals? The International Committee of Medical Journal Editors (ICMJE) recommends that journal editors publicly disclose their potential CoI [3]. However, a number of studies demonstrate that journal editors in general do not follow the policies they impose on authors and reviewers.

Studies presented in **Table 1** clearly show that the transparency of disclosure of editorial CoI has not improved across journals from a range of disciplines and influence in the scientific community in the last 12 years, despite greater awareness and the published evidence about the problem [3]. With the availability of the information on payments to physicians in the US through the Physician Sunshine Act from August 2013, it also became clear that physician-editors working as clinicians in the US received industry payments for their professional and research work [13,15-17]. This practice seems to be particularly common for high impact journals, both in general/internal medical and specialty disciplines (**Table 1**). The payments to editors varied a lot both between and within journals of different medical fields. For example, although the same percentage (28%) of physicians working as editors in 4 general/internal medicine journal received general payments (eg, consultancy, expert opinion, travel) from industry in 2014, the mean and the maximum payments received by editors of the *New England Journal of Medicine* and *JAMA* were 72 times (US$3899 vs US$54) and 100 times (US$78 617 vs US$795) higher that those received by editors of *JAMA Internal Medicine* and *Annals of Internal Medicine*, respectively [13]. 76% of cardiology journal editors and 56% of surgery journal editors received general industry payments, with the mean payment of US$225 556 and US$246, respectively, and the maximum payments of US$10 981 153 and US$1922, respectively [13]. Another analysis showed that 10%, 44% and 2% editors of internal medicine, cardiology and surgery journals with the highest number of citations in 2015 per specialty received more than US$10 000 as general payments from industry, respectively [17].

**Table 1:** Disclosures of editorial conflicts of interests (CoI) and payments to editors in biomedical journals*

Study authors, year	Journals included	Finding
**Declaration of CoI:**		
Cooper et al., 2006 [5]	91 high-impact general and specialty biomedical journals	40% of the journals stated that they had CoI policies for editors
Bhargava et al., 2007 [6]	12 gastroenterology and hepatology journals	17% of the journals publicly disclosed editorial CoI
Andraku et al., 2009 [7]	42 ophthalmology journals	5% of the journals publicly disclosed editorial CoI
Alfonso et al, 2012 [8]	45 European Society of Cardiology National Cardiovascular Journals	18% of the journals had a specific policy on editors’ CoI
Qureshi et al., 2012 [9]	15 gastroenterology and hepatology journals	33% of the journals publicly disclosed CoI policies for editors
Smith et al., 2012 [10]	10 high-impact medical journals	40% of the journals have easily accessible CoI policies for editors
Bosch et al., 2013 [11]	399 high-impact biomedical journals	39% of the journals required editors’ CoI disclosures
Broga et al., 2014 [12]	68 biomedical journals from Southeast and Eastern Europe	3% of the journals had CoI policies for editors
Liu et al., 2017 [13]	52 influential US medical journals from 25 specialties	33% of the journals had readily available editors’ CoI policies
Yang et al., 2017 [14]	30 Chinese-language and 37 English-language journals in China	No Chinese-language journals had CoI policies for editors; 50% of editorials in English-language journals had CoI disclosure
**Payments received by editors:**		
Liu et al., 2017 [13]	713 editors from 52 influential US medical journals from 25 specialties	51% of the editors received general and 19.5% research payments in 2014
Mehlman et al., 2017 [15]	15 orthopaedic surgery journals	4-73% of editorial board members received >US$10 000 in 2014
Verma, 2017 [16]	85 editorial board members from 3 US radiation oncology journals	76% of the editorial board members received payment in 2013-2015
Wong et al., 2017 [17]	333 editorial board members from 35 highly cited medical journals from 7 specialties	64% editorial board members received any industry-associated payments in 2013-2016

*Articles were identified in PubMed, Scopus, arXiv.org, PeerJ preprint and F1000 Resarch on 24 March 2018 by using the search key-words “conflict(s) of interest”, “competing interest(s)”, “declaration(s)” and “payment(s)” in combination with the key-word “editor(s)”

Why is disclosure of industry payments to editors relevant? Evidence shows that industry payments, even if they are modest, such as for meals, are associated with higher rates of prescription of brand-name medicines although generic drugs of similar efficacy are available, as well as greater expenditure on prescriptions per patient [18-21]. This means that editors who received industry payments, regardless of the amount, can make biased decisions, too, although sometimes in the opposite direction to the expected one [22]. On the other hand, the individuals do not like being considered biased, and mandating disclosure of potential CoI may be an incentive to avoid them [23].

The ICMJE states that any journal editor with a potential CoI should recuse himself or herself from editorial decisions affecting manuscripts that are considered for publication, especially when the editor is the author of the submitted work. The editor-in-chief must also know the potential CoI of the members of the editorial team and make them public on a regular basis [3]. Thus, the editorial CoI policy places the three main actors of the editorial process – authors, external reviewers and editors on the same level of the transparency demand. Unfortunately, the member journals of the ICMJE also do not follow well their own recommendations. We checked the availability of policies for declaring CoI for authors, reviewers and editors in the public domain, ie, at the journal web-pages, as well as the existence of public declaration of individual CoIs by journal editors (**Table 2**).

**Table 2:** Conflict of interest (CoI) policies of journal members of the International Committee of Journal Editors (ICMJE)*

Journal	Owner, country	CoI policy for:		Editors’ CoI declaration	
		**Authors**	**Reviewers**	**Policy**	**Individual declarations**
*Annals of Internal Medicine*	American College of Physicians, USA	Yes	Yes	No	No
*BMJ*	British Medical Association, UK	Yes	Yes	Yes	Yes
*Bulletin of the WHO*	World Health Organization, Switzerland	Yes	No	No	No
*Deutsches Ärzteblatt*	German Medical Association, Germany	Yes	No	No	No
*Ethiopian Journal of Health Sciences*	Jimma University, Ethiopia	No	No	No	No
*Iranian Journal of Medical Sciences*	Shiraz University of Medical Sciences, Iran	Yes	Yes	Yes	No
*JAMA*	American Medical Association, USA	Yes	No	Yes	No
*Journal of Korean Medical Science*	Korean Academy of Medical Sciences, Korean Medical Association, South Korea	Yes	No	No	No
*Lancet*	Elsevier, UK	Yes	No	No	No
*New England Journal of Medicine*	Massachusetts Medical Society, USA	Yes	Yes	Yes	No
*New Zealand Medical Journal*	New Zealand Medical Association, New Zealand	Yes	No	No	No
*PLOS Medicine*	Public Library of Science, USA	Yes	Yes	Yes	Yes
*Revista Médica de Chile*	Sociedad Médica de Santiago, Chile	Yes	No	No	No
*Ugeskrift for Laeger*	Danish Medical Association, Denmark	Yes	No	No	No

*Web pages of all ICMJE journals were searched in April 2018. For journals published in more than language, only the English version was searched.

While all 14 ICMJE member journals had detailed CoI declaration policies for authors, only 36% (5/14) had easily available policies for declaring reviewers’ CoIs, and those that use open peer review system (eg, *BMJ*) also publish CoI declaration for individual reviewers together with the relevant article. Only 36% (5/14) ICMJE member journals had publicly disclosed policies about managing editorial CoIs and 2 publicly posted declarations of current individual CoI for their editors. It is possible that those ICMJE-member journals that do not publicly disclose their editors’ individual CoI follow them internally when appropriate, but this would be against ICMJE recommendations for the transparency of CoI disclosures.

What can be done in the situation where we have so many good policies but so few actual application in practice? First, the ICMJE member journal should make sure that all recommended policies are fully implemented, so that they set real standards and examples for the editorial community. The policies on editorial CoI and declarations of individual CoIs for editors should be posted and easily identifiable on journal’s web pages. Transparency of editor’s CoI could be further increased by publishing individual editorial CoI declarations in the journal. In this way, such published item would be indexed in bibliographical databases, clearly visible and properly archived. Annual publication of editorial CoI declarations would ensure that possible changes are recorded or CoI declaration of new editors made public.

Publications of editorial CoI declaration is already the practice in some journals. **Table 3** presents the examples of editorial CoI declarations published as editorials or statements in journals and indexed in PubMed. It can be imagined that such declarations could be indexed with a specific tag, similar to those used to mark specific types of publications in MEDLINE [24], which could make them easily identifiable in bibliographical databases. In this way, disclosures of editorial CoI would reach the level of transparency required for all stakeholders in the publication process.

**Table 3:** Examples of individual conflict of interest declaration by journal editors indexed as separate bibliographical items in PubMed

**Journal reference**	**Statement if available as abstract in PubMed**
[No authors listed]. Financial disclosure for associate editors of the Cleveland Clinic Journal of Medicine. Cleve Clin J Med. 2010;77: 347.	–
[No authors listed]. Headache associate editors declaration of conflicts of interest. Headache. 2014;54:4-6.	–
Lubowitz JH. Editorial commentary: Editor's conflict of interest. Arthroscopy. 2015;31:1740.	The Editor-in-chief has recused himself from industry consulting, which he performed before assuming the position, and returned related royalties and divested related stock options, in order to mitigate against conflict-of-interest. The Editor discloses affiliation with an institution that receives support from diverse industry partners in support of research and education.
[No authors listed]. Conflict of Interest Declarations by Contributing Editors of the Special Issue on Early-Career Systems Microbiology Scientists, Sponsored by Janssen Human Microbiome Institute (JHMI). mSystems. 2018 Mar 6;3(2). pii: e00010-18.	–
Rey C, on behalf of Anales de Pediatría editorial team. Conflicts of Interest of the editors. (article in Spanish). An Pediatr (Barc). 2018;88:296-7.	–

**Correspondence to:** Ana Marušić *Journal of Global Health* ana.marusic@jogh.org
